# The Obesity–Bone Paradox in Postmenopausal Women: Divergent Associations of BMI with Bone Mineral Density and Trabecular Bone Score in a Real-World DXA Cohort

**DOI:** 10.3390/medicina62081555

**Published:** 2026-08-13

**Authors:** Robert Bot, Simona Daniela Cavalu

**Affiliations:** 1 Doctoral School of Biomedical Sciences, Faculty of Medicine and Pharmacy, University of Oradea, P-ta 1 Decembrie 10, 410073 Oradea, Romania; robertbbot@gmail.com; 2 Faculty of Medicine and Pharmacy, University of Oradea, P-ta 1 Decembrie 10, 410073 Oradea, Romania

**Keywords:** body mass index, obesity, bone mineral density, trabecular bone score, osteoporosis, postmenopausal women, DXA, bone microarchitecture

## Abstract

*Background and Objectives*: Body mass index (BMI) has a complex relationship with skeletal health. Higher BMI is often associated with higher bone mineral density (BMD), but this apparent densitometric advantage may not fully reflect bone microarchitecture. Trabecular bone score (TBS) may provide complementary information in overweight and obese postmenopausal women. This study evaluated the associations between BMI, BMD, and TBS in postmenopausal women and assessed whether increased BMI is associated with discordant skeletal phenotypes characterized by non-osteoporotic densitometric status but impaired trabecular microarchitecture. *Materials and Methods*: This retrospective observational study screened 110 DXA/TBS reports from patients evaluated in a tertiary DXA center. Inclusion criteria were female sex, postmenopausal status, available BMI or sufficient anthropometric data for BMI calculation, valid lumbar spine DXA, at least one valid hip DXA assessment, and valid lumbar TBS assessment. Four cases were excluded: two male patients and two non-menopausal female patients. The final analytical cohort included 106 postmenopausal women. Lumbar spine and hip BMD, T-scores, TBS, Bone Resilience Index, anthropometric variables, and menopausal variables were extracted from DXA/TBS reports. BMI was analyzed both continuously and by category: under/normal-weight, overweight, and obesity. Correlation analyses, group comparisons, and linear regression models were used to evaluate the relationship between BMI and skeletal parameters. Discordant skeletal phenotypes were defined descriptively as non-osteoporotic DXA findings combined with degraded TBS. *Results*: The final cohort had a mean age of 65.2 ± 7.6 years and a mean BMI of 27.3 ± 4.5 kg/m^2^. BMI was positively associated with lumbar spine BMD (r = 0.392, *p* < 0.001), minimum femoral neck BMD (r = 0.382, *p* < 0.001), and minimum total hip BMD (r = 0.508, *p* < 0.001), but negatively associated with TBS (r = −0.307, *p* = 0.001). Mean TBS decreased across BMI categories, from 1.283 ± 0.084 in the under/normal-weight group to 1.255 ± 0.088 in the overweight group and 1.199 ± 0.109 in the obesity group. In the fully adjusted regression model including BMI, age, years since menopause, lumbar spine BMD, and lowest hip T-score, BMI remained an independent negative predictor of TBS (β = −0.0126, *p* < 0.001). Discordant skeletal phenotypes combining non-osteoporotic DXA status with degraded TBS were more frequent in patients with obesity. *Conclusions*: In postmenopausal women, higher BMI was associated with higher BMD but lower TBS, supporting a divergent relationship between densitometric findings and DXA-derived trabecular texture parameters. These findings suggest that TBS may complement BMD in describing skeletal imaging status in overweight and obese women. Because fracture outcomes and complete clinical risk data were not available, the present results should be interpreted as evidence of imaging discordance rather than direct evidence of clinical underdiagnosis or misclassification.

## 1. Introduction

Osteoporosis remains one of the major skeletal disorders affecting postmenopausal women, with bone fragility resulting from the combined effects of reduced bone mass, microarchitectural deterioration, altered skeletal properties, and increased susceptibility to fracture. Dual-energy X-ray absorptiometry (DXA) remains the reference method for the diagnosis of osteoporosis in clinical practice, and T-score thresholds continue to provide the operational framework for classifying normal bone mineral density (BMD), osteopenia, and osteoporosis [1,2,3,4,5,6]. However, BMD reflects only one component of skeletal strength and does not fully capture microarchitectural deterioration or other qualitative determinants of fracture risk [1,2].

Trabecular bone score (TBS) has emerged as a non-invasive analytical parameter derived from lumbar spine DXA images, providing indirect information regarding trabecular microarchitecture [2,3,4]. Lower TBS values have been associated with increased fracture risk independently of BMD, and TBS has been incorporated into fracture risk assessment frameworks as a complementary tool to densitometric evaluation [5,6]. Current clinical positions support the use of TBS as an adjunct to BMD, particularly in situations where densitometric findings may not fully reflect skeletal fragility [3,4,7,8].

Body mass index (BMI) has a complex relationship with skeletal health. BMI was selected as the primary anthropometric indicator because it is routinely available in clinical DXA reports, is easily calculated from standard height and weight measurements, and remains widely used in epidemiological and clinical studies evaluating obesity-related skeletal health. However, BMI is an indirect marker of body size and adiposity and cannot distinguish between fat mass, lean mass, visceral adiposity, fat distribution, or sarcopenic obesity. Therefore, BMI should not be interpreted as a direct measure of body composition, but rather as a pragmatic clinical anthropometric index available in routine DXA/TBS assessment. Higher BMI is frequently associated with higher BMD, particularly at weight-bearing skeletal sites, most likely through greater mechanical loading and related adaptive skeletal responses [9,10,11,12]. This apparent densitometric advantage has historically contributed to the perception that obesity may be protective against osteoporosis. Nevertheless, epidemiological and clinical studies have shown that obesity does not uniformly protect against fractures and may be associated with site-specific fracture risk, altered skeletal properties, impaired trabecular microarchitecture, altered fall mechanics, metabolic dysfunction, chronic low-grade inflammation, adipokine imbalance, sarcopenia, and diabetes-related skeletal changes [13,14,15,16,17,18,19,20,21].

This apparent contradiction has led to the concept of an obesity–bone paradox: patients with increased BMI may present relatively preserved or even increased BMD while quality still showing impaired trabecular microarchitecture, altered skeletal properties, or increased skeletal fragility. In this context, TBS may be particularly relevant, because several studies have reported an inverse association between BMI and TBS despite positive or neutral associations between BMI and BMD [22,23,24,25,26,27,28]. These findings suggest that, in overweight and obese postmenopausal women, BMD alone may overestimate skeletal integrity if microarchitectural deterioration is not simultaneously assessed.

Although the inverse association between BMI and TBS has been reported previously, its clinical interpretation in routine DXA practice remains incompletely characterized, particularly in relation to patients whose BMD does not meet densitometric osteoporosis thresholds. The present study contributes to the existing literature by evaluating this issue in a Romanian real-world DXA/TBS cohort of postmenopausal women, by using bilateral hip measurements when available and minimum hip values for densitometric classification, and by characterizing discordant skeletal phenotypes in which non-osteoporotic DXA findings coexist with degraded TBS. Thus, the study was intended not simply to confirm the BMI–BMD–TBS relationship but to provide a clinically oriented description of imaging discordance across BMI categories.

The primary objective was to assess the relationship between BMI, BMD, and TBS. Secondary objectives were to compare skeletal parameters across BMI categories, evaluate whether BMI remains independently associated with TBS after adjustment for age, menopausal duration, and BMD, and identify discordant skeletal phenotypes in which non-osteoporotic DXA findings coexist with degraded TBS.

## 2. Materials and Methods

### 2.1. Study Design and Setting

This study was designed as a retrospective observational analysis of DXA/TBS reports from patients evaluated in a tertiary DXA center in Oradea, Romania. The analysis was designed to investigate the relationship between body mass index (BMI), bone mineral density (BMD), and trabecular bone score (TBS), with particular emphasis on the potential divergence between densitometric and microarchitectural skeletal parameters.

All DXA/TBS reports were anonymized before analysis. Each report was assigned a study code according to the source file number, from P001 to P110. No directly identifiable patient information was included in the analytical dataset.

### 2.2. Eligibility Criteria and Study Population

A total of 110 DXA/TBS reports were screened for eligibility. The inclusion criteria were: female sex, postmenopausal status, available age, available height and weight or directly reported BMI, valid lumbar spine DXA assessment, at least one valid hip DXA assessment, and valid lumbar TBS assessment.

The final TBS inclusion criterion was defined as valid lumbar TBS assessment available, calculated on the vertebral levels accepted by the TBS software after exclusion of non-evaluable vertebrae, when applicable. Therefore, reports were considered eligible if the TBS software provided a valid lumbar TBS value, even when the calculation did not include all L1–L4 vertebrae because of vertebral exclusion.

The exclusion criteria were: male gender, non-menopausal status, missing or non-calculable BMI, missing lumbar TBS, missing valid lumbar spine DXA, absence of valid hip DXA assessment, incomplete or unreadable report, and major technical invalidation preventing reliable interpretation of DXA or TBS parameters.

**Figure 1:**
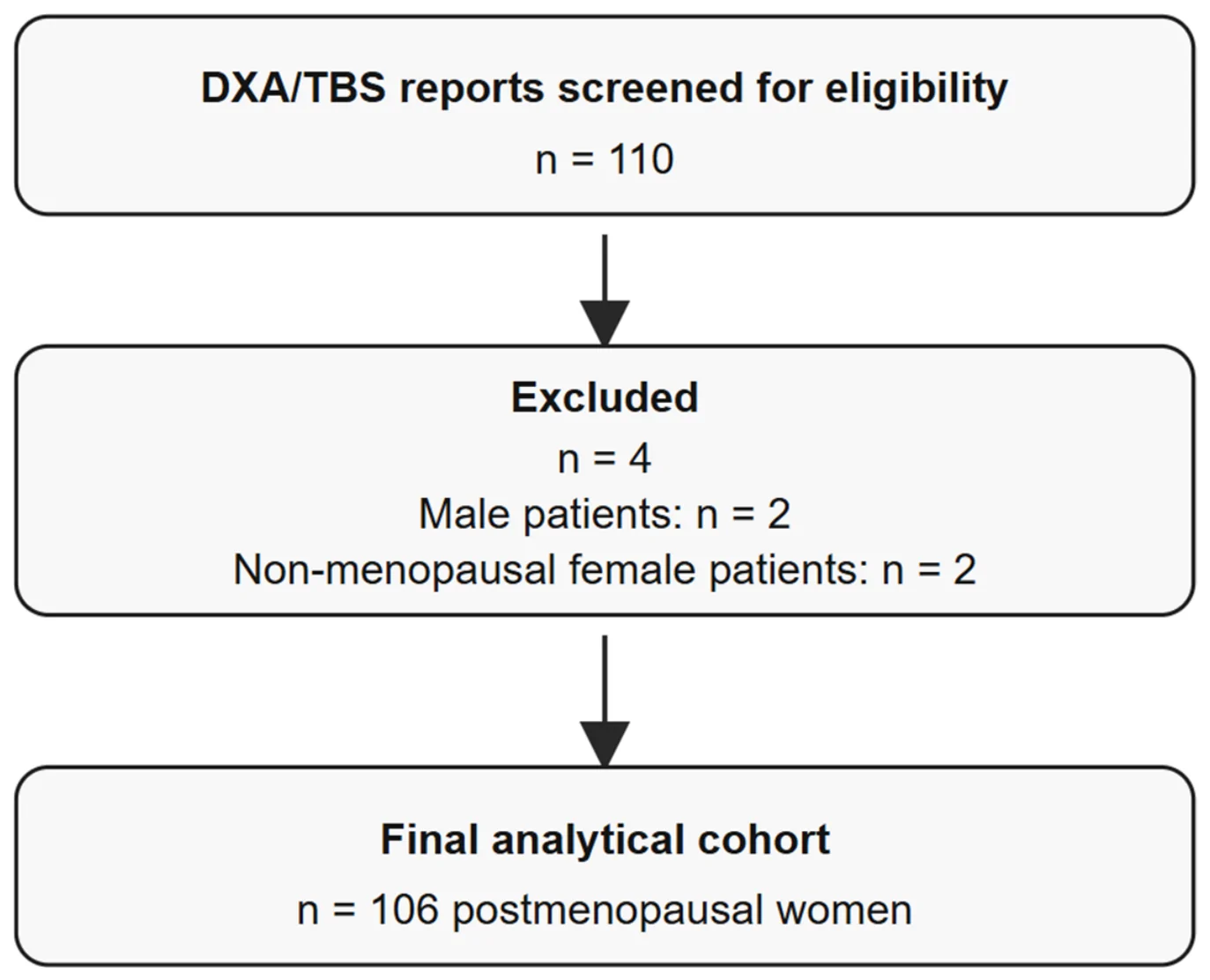
Flow diagram of participants selection. DXA acquisition and densitometric assessment.

After screening, four cases were excluded: two male patients and two non-postmenopausal females. The final analytical cohort included 106 postmenopausal women, as presented in Figure 1.

DXA examinations were performed using a Hologic Horizon Wi densitometer (Hologic Inc., Marlborough, MA, USA), with DXA analysis software version 13.6.1.3:5. Lumbar spine and bilateral hip scans were reviewed when available. Extracted densitometric variables included lumbar spine BMD and T-score, femoral neck BMD and T-score, total hip BMD and T-score, and the corresponding densitometric diagnostic categories.

Densitometric classification was based on standard T-score thresholds: normal BMD was defined as T-score ≥ −1.0, osteopenia as T-score between −1.0 and −2.5, and osteoporosis as T-score ≤ −2.5 [29,30,31,32]. Overall DXA category was defined as the most severe classification identified across lumbar spine and hip sites.

For hip analyses, bilateral measurements were retained when available. Minimum femoral neck BMD and T-score were defined as the lower value between the left and right femoral neck. Minimum total hip BMD and T-score were defined as the lower value between the left and right total hip. The lowest hip T-score was defined as the lowest T-score among available femoral neck and total hip measurements bilaterally.

### 2.3. Trabecular Bone Score and Bone Resilience Index

Lumbar TBS was extracted from the DXA/TBS reports. TBS values were generated using TBS iNsight software version 3.1.2. The direct soft-tissue thickness correction implemented in TBS version 4.0 was not available for these reports and was therefore not applied. TBS was analyzed both as a continuous variable and as a categorical variable. TBS categories were retained according to the thresholds used by the reporting software and classified as normal, partially degraded, or degraded. TBS was interpreted as a DXA-derived surrogate marker of trabecular microarchitecture, complementary to BMD, and not as a direct measurement of bone quality [2,3,4,5,6,7,8].

When the TBS report specified the vertebral region used for calculation, this information was recorded. Cases with vertebral-level exclusions were retained if the software provided a valid lumbar TBS value based on the accepted vertebral levels.

Bone Resilience Index (BRI), when available, was extracted as a categorical parameter and retained according to the software-reported classification. Low or severely low BRI was considered an unfavorable bone resilience profile. BRI was included as a descriptive software-derived parameter from the DXA/TBS reports and was not considered a primary outcome of the present analysis.

### 2.4. Anthropometric and Menopausal Variables

Age, height, weight, BMI, age at menopause, and years since menopause were extracted from the DXA reports. When years since menopause was not directly reported, it was calculated as chronological age minus age at menopause.

BMI was analyzed both as a continuous and categorical variable. BMI categories were defined according to standard WHO thresholds: underweight, BMI < 18.5 kg/m^2^; normal-weight, BMI 18.5–24.9 kg/m^2^; overweight, BMI 25.0–29.9 kg/m^2^; and obesity, BMI ≥ 30.0 kg/m^2^ [33]. Because only one participant was reported as underweight, underweight and normal-weight participants were combined into a single under/normal-weight group for comparative analyses. The final BMI groups were therefore under/normal-weight, overweight, and obesity.

Although DXA can provide detailed body composition parameters, including total fat mass, lean mass, android/gynoid fat distribution, and visceral adiposity estimates, these variables were not systematically available in the retrospective clinical DXA/TBS reports analyzed in this study. Therefore, body composition parameters could not be included in the statistical analyses, and BMI was retained as the only consistently available anthropometric indicator.

### 2.5. Definition of Discordant Skeletal Phenotypes

Several clinically relevant discordant skeletal phenotypes were predefined. Non-osteoporotic DXA status was defined as normal BMD or osteopenia, without densitometric osteoporosis. A discordant skeletal phenotype was defined as non-osteoporotic DXA status combined with degraded TBS. These categories were considered imaging-defined discordant phenotypes and not validated clinical risk categories, because fracture history, complete FRAX probabilities, and longitudinal fracture outcomes were not systematically available.

Additional exploratory discordant phenotypes included non-osteoporotic DXA status combined with partially degraded or degraded TBS, non-osteoporotic hip DXA status combined with degraded TBS, and non-osteoporotic hip DXA status combined with partially degraded or degraded TBS. These phenotypes were evaluated across BMI categories to determine whether overweight and obesity were associated with a higher prevalence of trabecular impairment despite non-osteoporotic densitometric findings.

### 2.6. Statistical Analysis

Continuous variables were summarized as mean ± standard deviation. Categorical variables were summarized as counts and percentages. Comparisons across BMI categories were performed using one-way analysis of variance for continuous variables. Kruskal–Wallis testing was used as a non-parametric sensitivity analysis when appropriate. Categorical variables were compared using Pearson chi-square tests or Fisher exact tests, depending on expected cell counts.

Pearson correlation coefficients were calculated to evaluate associations between BMI and continuous skeletal parameters, including TBS, lumbar spine BMD, minimum femoral neck BMD, minimum total hip BMD, and corresponding T-score variables. Correlations with age and years since menopause were also assessed.

Linear regression models were used to evaluate the association between BMI and TBS. First, an unadjusted model was fitted with TBS as the dependent variable and BMI as the independent variable. A second model adjusted for age and years since menopause. A fully adjusted model included BMI, age, years since menopause, lumbar spine BMD, and lowest hip T-score. Regression coefficients, 95% confidence intervals, *p* values, and model R^2^ values were reported.

Regression diagnostics were performed for the linear regression models. The approximate normality of residuals was evaluated by visual inspection of residual histograms and Q–Q plots, and homoscedasticity was assessed using residual-versus-fitted plots. Influential observations were screened using leverage and Cook’s distance. Multicollinearity was assessed using variance inflation factors (VIF). Lumbar spine BMD was included in the fully adjusted model because TBS is derived from lumbar spine DXA images, whereas lowest hip T-score was included to account for clinically relevant hip densitometric status based on the lowest available bilateral hip measurement.

For ordinal patterns across BMI categories, BMI group was treated as an ordered variable. Trend analyses were used to evaluate whether increasing BMI category was associated with progressive worsening of TBS category or higher prevalence of predefined discordant risk phenotypes.

To emphasize estimation precision rather than statistical significance alone, 95% confidence intervals were reported for correlation coefficients and regression coefficients. For key subgroup proportions describing TBS impairment and discordant skeletal phenotypes, Wilson 95% confidence intervals were calculated to indicate the uncertainty of prevalence estimates, particularly in analyses involving small BMI subgroups.

A two-sided *p* value < 0.05 was considered statistically significant.

### 2.7. Ethical Considerations

The study was conducted in accordance with the Declaration of Helsinki, and approved by the Institutional Review Board/Ethical Council of the Emergency County Clinical Hospital, Oradea, Bihor, Romania (no. 39135/9 November 2023 and 39657/15 November 2023) and Ethical Committee of University of Oradea, Faculty of Medicine and Pharmacy (01/20 December 2023). This retrospective observational study used anonymized DXA/TBS report data from patients evaluated in a tertiary DXA center. No additional clinical procedures were performed for the purpose of this study, and no directly identifiable patient information was included in the analytical dataset. Each report was anonymized and assigned a study code before data extraction and statistical analysis.

## 3. Results

### 3.1. Study Population

A total of 110 DXA/TBS reports were screened for eligibility. Four cases were excluded: two male patients and two non-menopausal females. The final analytical cohort included 106 postmenopausal women with valid DXA and lumbar TBS assessments.

The mean age of the cohort was 65.2 ± 7.6 years, the mean BMI was 27.3 ± 4.5 kg/m^2^, and the mean time since menopause was 17.5 ± 8.3 years.

Participants were stratified into three BMI categories. Underweight and normal-weight patients were combined into a single under/normal-weight group, as only one participant was identified as underweight. The final BMI strata were: under/normal-weight, n = 36; overweight, n = 37; and obesity, n = 33.

The clinical, anthropometric, densitometric, and TBS characteristics of the cohort according to BMI category are presented in Table 1.

According to the overall DXA classification, 5 patients (4.7%) had normal BMD, 47 (44.3%) had osteopenia, and 54 (50.9%) had osteoporosis. Regarding trabecular bone score, 29 patients (27.4%) had normal TBS, 33 (31.1%) had partially degraded TBS, and 44 (41.5%) had degraded TBS.

### 3.2. BMI Categories and Skeletal Parameters

BMI categories showed divergent associations with densitometric and microarchitectural parameters. Lumbar spine BMD increased progressively across BMI groups, from 0.768 ± 0.128 g/cm^2^ in the under/normal-weight group to 0.788 ± 0.091 g/cm^2^ in the overweight group and 0.870 ± 0.160 g/cm^2^ in the obesity group. A similar pattern was observed for hip parameters, with higher minimum femoral neck BMD and minimum total hip BMD in participants with obesity.

In contrast, TBS decreased with increasing BMI. Mean TBS was 1.283 ± 0.084 in the under/normal-weight group, 1.255 ± 0.088 in the overweight group, and 1.199 ± 0.109 in the obesity group. This pattern indicates a discordant skeletal profile, in which higher BMI was associated with higher BMD but lower trabecular bone score.

**Figure 2:**
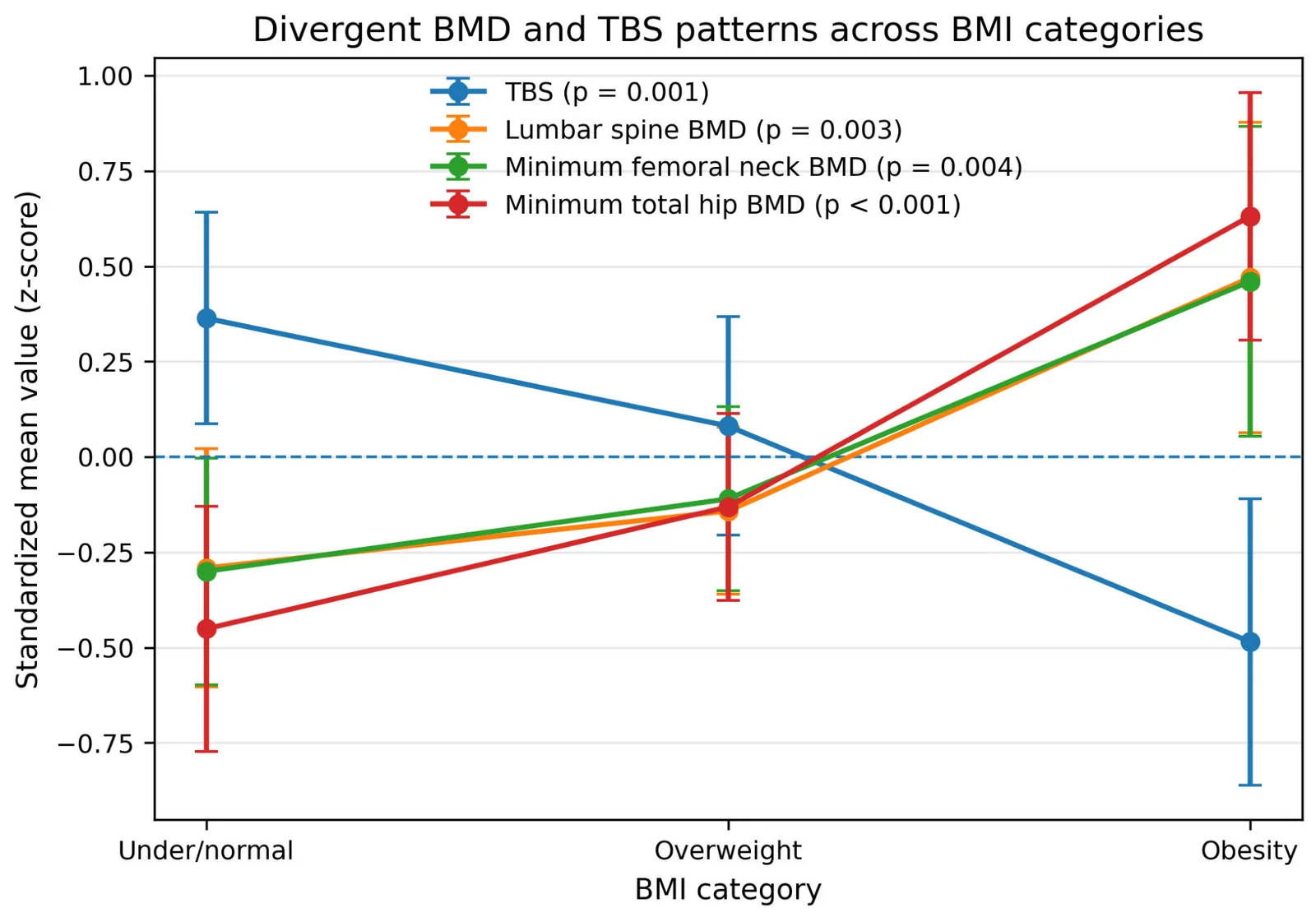
Standardized densitometric and microarchitectural parameters across BMI categories. Values are shown as standardized means, and error bars indicate 95% confidence intervals. Lumbar spine BMD, minimum femoral neck BMD, and minimum total hip BMD increased across BMI categories, whereas TBS showed an opposite downward pattern.

This divergent pattern is illustrated in Figure 2, where standardized densitometric parameters increased across BMI categories, whereas TBS showed an opposite downward trend.

**Figure 3:**
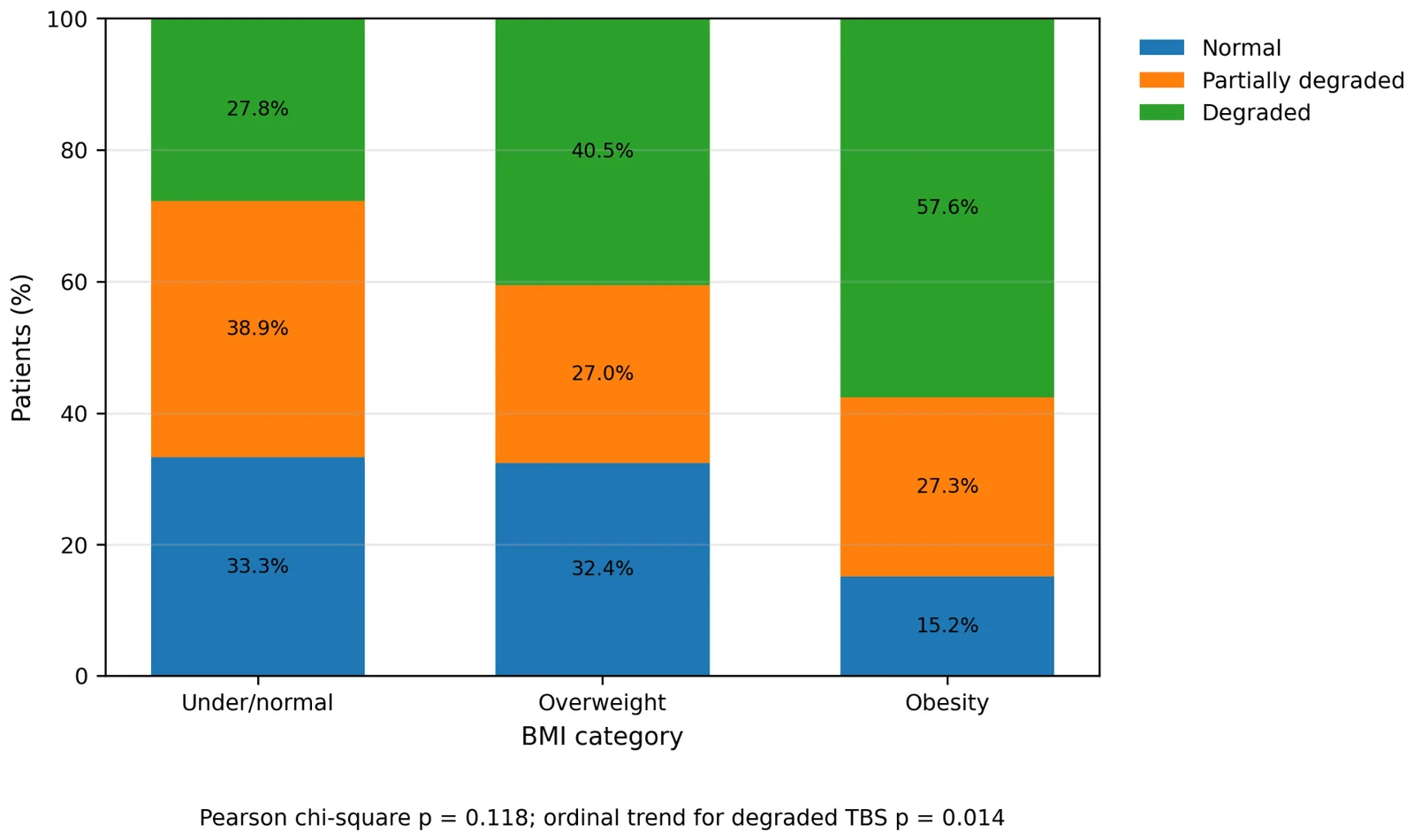
TBS category distribution across BMI categories. The figure shows the percentage distribution of normal, partially degraded, and degraded TBS across BMI groups. The full three-category TBS distribution showed Pearson chi-square *p* = 0.118, while the trend analysis for increasing prevalence of degraded TBS across ordered BMI categories showed *p* = 0.014.

The distribution of TBS categories across BMI groups is shown in Figure 3. Although the full three-category TBS distribution showed only a moderate difference across BMI strata, the proportion of degraded TBS increased progressively from the under/normal-weight group to the obesity group.

### 3.3. Correlation Analysis

Correlation analyses confirmed the divergent associations between BMI, BMD, and TBS. BMI was inversely associated with TBS, while positive associations were observed between BMI and lumbar spine BMD, minimum femoral neck BMD, minimum total hip BMD, and hip T-score parameters.

The correlations between BMI and densitometric or microarchitectural parameters are summarized in Table 2.

**Figure 4:**
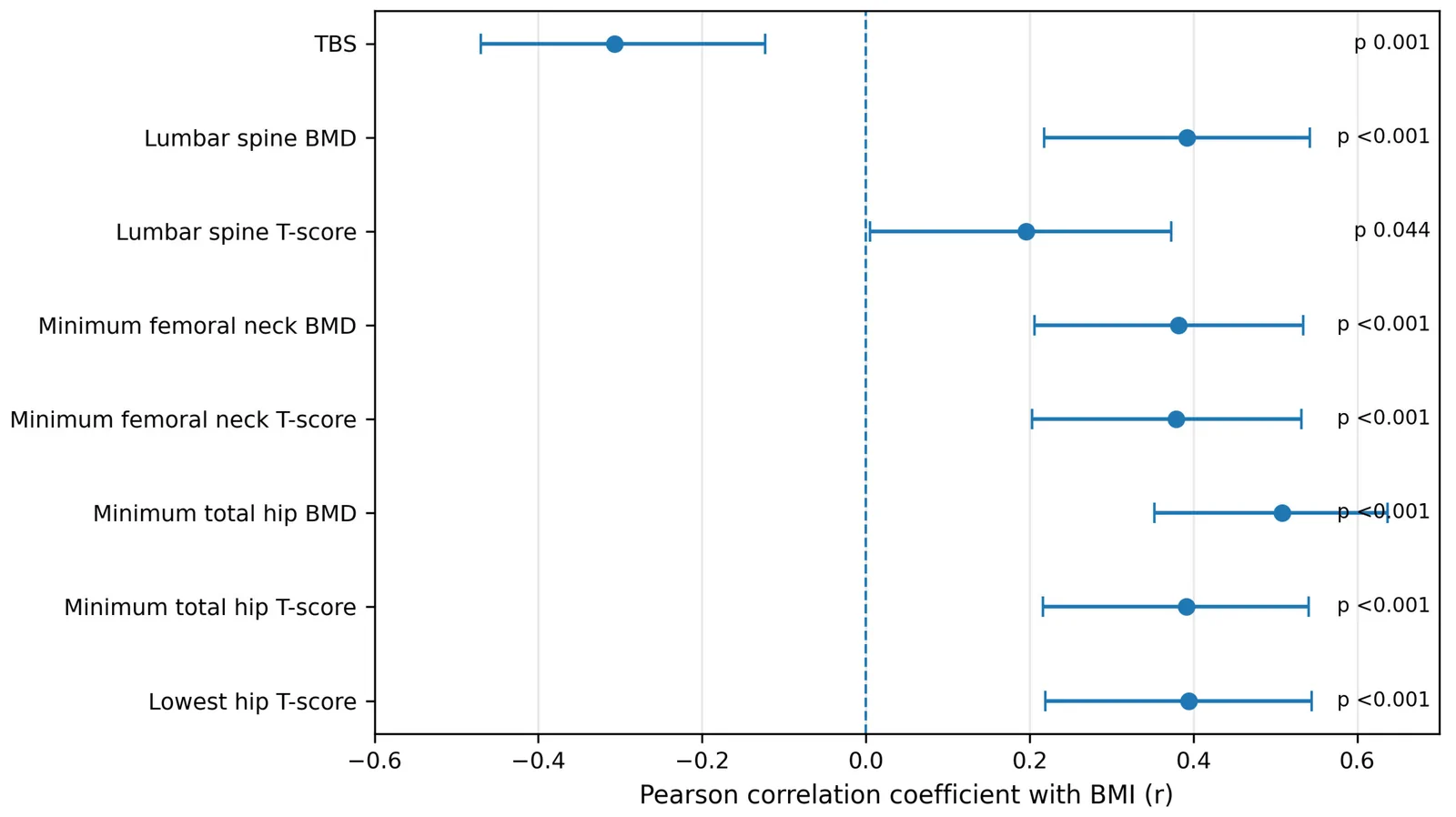
Associations between BMI and skeletal parameters. Pearson correlation coefficients are shown with 95% confidence intervals. BMI was inversely associated with TBS (r = −0.307; 95% CI, −0.470 to −0.123; *p* = 0.001) and positively associated with densitometric parameters, particularly minimum total hip BMD (r = 0.508; 95% CI, 0.352 to 0.637; *p* < 0.001).

Association between BMI and TBS is further shown in Figure 4.

BMI was negatively correlated with TBS (r = −0.307; *p* = 0.001). Conversely, BMI was positively correlated with lumbar spine BMD (r = 0.392; *p* < 0.001), minimum femoral neck BMD (r = 0.382; *p* < 0.001), and minimum total hip BMD (r = 0.508; *p* < 0.001). BMI was not significantly associated with age or years since menopause.

### 3.4. Regression Analysis

Linear regression models were used to evaluate whether BMI remained associated with TBS after adjustment for relevant clinical and densitometric parameters. Regression diagnostics did not indicate relevant multicollinearity in the fully adjusted model. VIF values were 1.29 for BMI, 2.62 for age, 2.41 for years since menopause, 1.65 for lumbar spine BMD, and 1.92 for lowest hip T-score. These values indicated acceptable collinearity among predictors.

The regression models are presented in Table 3.

In the unadjusted model, BMI was significantly and negatively associated with TBS. Each 1 kg/m^2^ increase in BMI was associated with a mean decrease of 0.0068 in TBS. This association remained significant after adjustment for age and years since menopause.

In the multivariable model, which included BMI, age, years since menopause, lumbar spine BMD, and lowest hip T-score, BMI remained negatively associated with TBS. However, this model should be interpreted as a partially adjusted model, because several clinically relevant confounders were not available in the retrospective DXA/TBS reports. Lumbar spine BMD was positively associated with TBS in the same model, whereas age, years since menopause, and lowest hip T-score were not significant independent predictors. The fully adjusted model explained 43.6% of the variance in TBS.

### 3.5. TBS Impairment and Discordant Skeletal Phenotypes Across BMI Categories

Several TBS impairment patterns and discordant skeletal phenotypes were identified across BMI categories. These predefined imaging phenotypes were used to evaluate whether patients with higher BMI were more likely to show impaired trabecular microarchitecture despite non-osteoporotic densitometric findings. Because clinical fracture outcomes were not available, these categories should be interpreted as descriptive imaging phenotypes rather than validated clinical risk categories.

The distribution of discordant microarchitectural risk phenotypes across BMI categories is presented in Table 4.

Degraded TBS was present in 44 patients (41.5%) overall. Its prevalence increased progressively across BMI strata: 10 of 36 patients in the under/normal-weight group (27.8%; 95% CI, 15.8–44.0%), 15 of 37 in the overweight group (40.5%; 95% CI, 26.3–56.5%), and 19 of 33 in the obesity group (57.6%; 95% CI, 40.8–72.8%). Because these subgroup estimates were based on relatively small cell counts, they should be interpreted as exploratory prevalence estimates rather than definitive subgroup effects.

A discordant skeletal phenotype characterized by non-osteoporotic overall DXA classification but degraded TBS was identified in 19 patients (17.9%). This phenotype was more frequent with increasing BMI, occurring in 2 of 36 patients in the under/normal-weight group (5.6%; 95% CI, 1.5–18.1%), 6 of 37 in the overweight group (16.2%; 95% CI, 7.7–31.1%), and 11 of 33 in the obesity group (33.3%; 95% CI, 19.8–50.4%).

The same pattern was more pronounced when hip DXA status was considered. A non-osteoporotic hip DXA classification combined with degraded TBS was present in 32 patients (30.2%) overall, including 5 of 36 patients in the under/normal-weight group (13.9%; 95% CI, 6.1–28.7%), 10 of 37 in the overweight group (27.0%; 95% CI, 15.4–43.0%), and 17 of 33 in the obesity group (51.5%; 95% CI, 35.2–67.5%).

**Figure 5:**
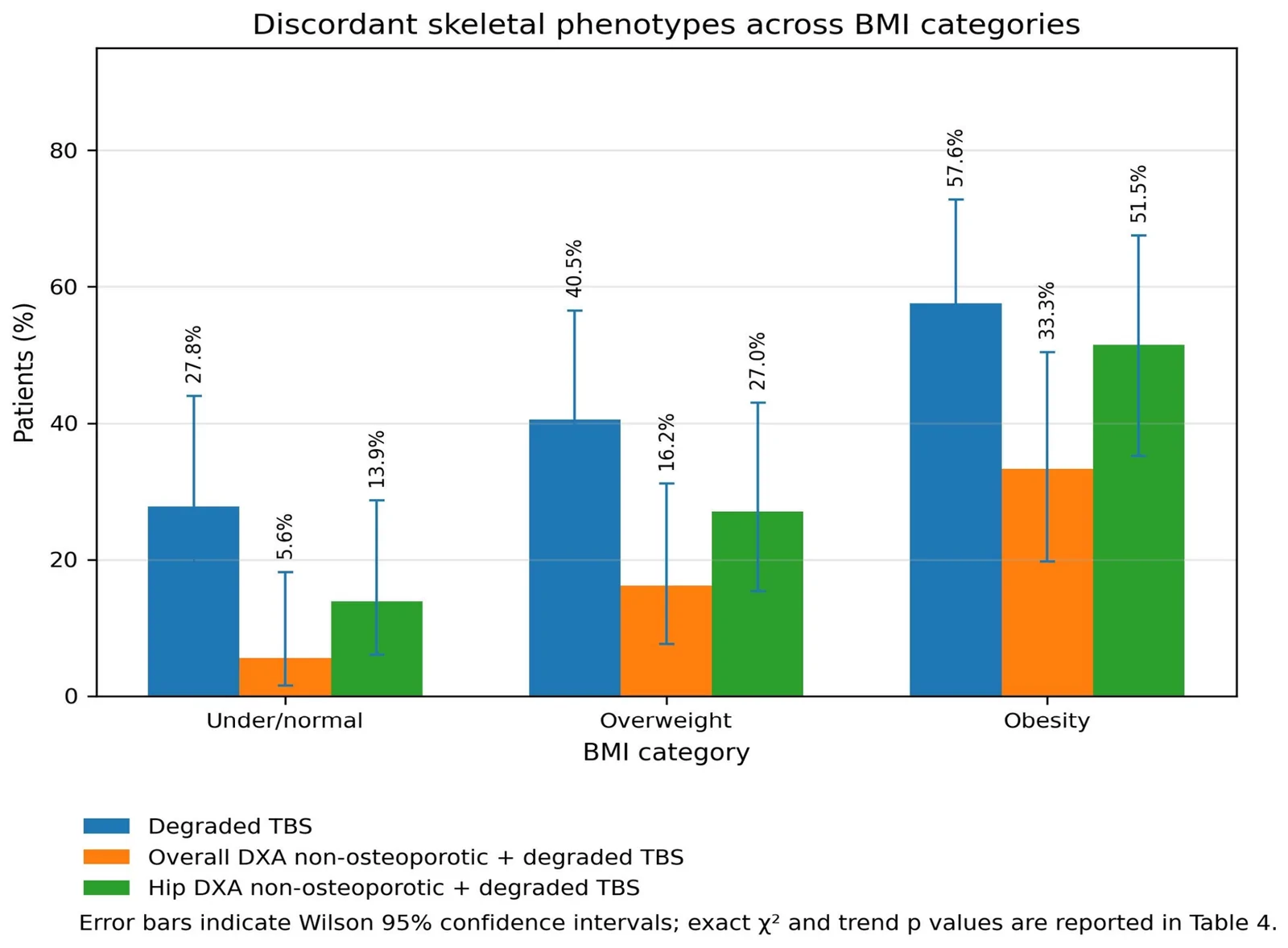
Discordant skeletal phenotypes across BMI categories. Bars represent subgroup proportions and error bars indicate Wilson 95% confidence intervals. Exact chi-square *p* values and trend *p* values for each phenotype are reported in Table 4.

The progressive increase in discordant microarchitectural risk phenotypes across BMI categories is illustrated in Figure 5.

When partially degraded and degraded TBS categories were combined, discordant microarchitectural phenotypes became even more frequent, particularly among patients with obesity. These findings indicate that increased BMI may be associated with apparently more favorable densitometric parameters while simultaneously being linked to impaired trabecular microarchitecture.

## 4. Discussion

### 4.1. Principal Findings

In this real-world cohort of postmenopausal women undergoing DXA and TBS assessment, BMI showed divergent associations with densitometric and microarchitectural skeletal parameters. Higher BMI was associated with higher lumbar spine, femoral neck, and total hip BMD, but with lower TBS. This divergence persisted in correlation analyses and remained evident after adjustment for age, years since menopause, lumbar spine BMD, and lowest hip T-score. These findings support the concept that increased BMI may be associated with an apparently favorable densitometric profile while simultaneously being linked to impaired trabecular microarchitecture.

These findings should be interpreted as confirmatory with respect to the previously described divergent BMI–BMD–TBS relationship. The added value of the present study lies in its real-world Romanian DXA/TBS setting, the use of minimum hip values derived from bilateral hip measurements when available, and the clinically interpretable characterization of discordant skeletal phenotypes across BMI categories.

The main clinical implication of this study is that BMD alone may not fully characterize skeletal status in overweight and obese postmenopausal women. In particular, discordant phenotypes combining non-osteoporotic DXA findings with degraded TBS were more frequent among patients with obesity. This suggests that, in patients with higher BMI, trabecular microarchitectural assessment may provide clinically relevant information beyond areal BMD.

The Bone Resilience Index was retained as a descriptive software-derived parameter because it was available in the DXA/TBS reports. However, BRI did not show a significant distributional difference across BMI categories and was not used as a primary endpoint. Therefore, the interpretation of the present study is based primarily on BMD, TBS, and predefined discordant skeletal phenotypes.

### 4.2. BMI and BMD: Apparent Densitometric Protection

The positive association between BMI and BMD observed in the present study is consistent with previous evidence showing that higher body weight and BMI are frequently associated with higher BMD, particularly at weight-bearing skeletal sites [9,10,11,12]. This relationship is commonly attributed to increased mechanical loading, which may stimulate adaptive skeletal responses and contribute to higher measured areal BMD. In our cohort, minimum total hip BMD and minimum femoral neck BMD increased progressively across BMI categories, and lumbar spine BMD was also higher among patients with obesity.

However, the interpretation of higher BMD in obesity requires caution. Although increased BMI may be associated with greater BMD, obesity is not uniformly protective against fracture [13,14,16,17]. Large observational studies and meta-analyses have shown that fracture risk in obesity is complex, site-specific, and influenced by factors beyond BMD, including fall mechanics, muscle function, metabolic comorbidities, chronic inflammation, adiposity distribution, and diabetes-related alterations in skeletal material or microarchitectural properties [13,14,16,17,18,19,20,21]. Therefore, higher BMD in patients with obesity should not automatically be interpreted as equivalent to better skeletal strength.

### 4.3. BMI and TBS: Evidence of Microarchitectural Impairment

In contrast to the positive association with BMD, BMI was negatively associated with TBS. This is the central finding of the present analysis. The inverse relationship between BMI and TBS is consistent with previous studies reporting lower TBS values in individuals with higher BMI, even when BMD is preserved or increased [22,23,24,25,26,27,28]. In our cohort, mean TBS decreased progressively from the under/normal-weight group to the obesity group, and BMI remained negatively associated with TBS in multivariable regression. Because these clinical and metabolic variables were not systematically available, the observed association between higher BMI and lower TBS may partly reflect unmeasured comorbidity, treatment exposure, lifestyle factors, or metabolic risk profiles rather than BMI alone.

This divergence between BMD and TBS supports the concept of an obesity–bone paradox. While BMD may capture the densitometric effects of higher body mass and mechanical loading, TBS may reflect aspects of trabecular texture and microarchitectural deterioration that are not captured by areal BMD alone [2,3,4,5,6,7,8]. Thus, the combination of higher BMD and lower TBS in patients with obesity may indicate a discrepancy between areal bone density and DXA-derived trabecular texture parameters, rather than a directly measured difference in bone quality.

Several mechanisms described in the literature may potentially contribute to the divergent relationship between BMI, BMD, and TBS, including chronic low-grade inflammation, altered adipokine secretion, insulin resistance, ectopic fat deposition, reduced mobility, sarcopenic obesity, visceral adiposity, and type 2 diabetes-related skeletal changes [18,19,20,21,34,35,36,37]. However, these variables were not measured in the present study. Therefore, these mechanisms should be interpreted as possible explanations for the observed imaging pattern rather than as conclusions directly supported by the present data. Because detailed metabolic, inflammatory, and body composition parameters were unavailable, the observed inverse association between BMI and TBS may reflect biological skeletal effects, technical influences related to soft tissue thickness, unmeasured comorbidity, or a combination of these factors.

The interpretation of BMI-related skeletal findings should also consider the heterogeneity of body composition. Individuals with similar BMI may have markedly different proportions of fat mass and lean mass, different patterns of fat distribution, and different degrees of visceral adiposity or sarcopenic obesity. These differences may influence BMD and DXA-derived trabecular texture parameters in different ways. Higher lean mass may contribute to higher BMD through mechanical loading and muscle–bone interactions, whereas excess adiposity, particularly visceral or metabolically unfavorable adiposity, may be associated with inflammatory, endocrine, and metabolic alterations that could adversely affect skeletal fragility-related properties. Therefore, the finding that BMI was positively associated with BMD but negatively associated with TBS should be interpreted in the context of body composition heterogeneity rather than as a uniform effect of obesity. Previous studies investigating body composition and skeletal assessment have also emphasized that body composition parameters may provide clinically relevant information beyond BMI alone.

For example, Sulis et al. reported sex- and obesity-specific associations between body composition parameters and ultrasound-assessed radial velocity of sound, supporting the relevance of fat mass, lean mass, and obesity status in skeletal assessment. In addition, Vorobeľová et al. showed that reproductive, socio-demographic, and lifestyle-related factors may contribute to obesity-related health outcomes in women, further supporting the need to interpret BMI within a broader biological and clinical context [38,39].

### 4.4. Clinical Relevance of Discordant Skeletal Phenotypes

A clinically relevant observation was the increasing prevalence of discordant skeletal phenotypes across BMI categories. Patients with obesity more frequently showed non-osteoporotic DXA status combined with degraded TBS. This pattern was especially evident when hip DXA status was considered: a substantial proportion of obese patients had non-osteoporotic hip DXA findings but degraded TBS.

However, these phenotypes should be interpreted as imaging-defined discordance rather than validated fracture-risk categories. The present study did not include systematic fracture history, complete FRAX probabilities, or longitudinal fracture incidence; therefore, it cannot determine whether these discordant skeletal phenotypes translate into higher clinical fracture risk.

This finding is clinically relevant because hip BMD is often strongly influenced by body weight and mechanical loading. In patients with higher BMI, hip BMD may appear relatively preserved while TBS may be degraded. However, in the absence of systematic fracture history, complete FRAX probabilities, or longitudinal fracture outcomes, this pattern should not be interpreted as direct evidence of clinical misclassification or underdiagnosis. Rather, it demonstrates imaging discordance between densitometric status and DXA-derived trabecular texture parameters.

Therefore, TBS may be useful as a complementary parameter in overweight and obese postmenopausal women whose BMD does not meet densitometric osteoporosis thresholds, but its interpretation should be integrated with the overall clinical risk profile rather than used as a standalone indicator of fracture risk.

The present findings do not imply that TBS should replace BMD. Rather, they support the complementary interpretation of BMD and TBS. BMD remains the diagnostic reference for osteoporosis and an essential component of fracture risk assessment [29,30,31,32,40,41]. TBS adds information related to trabecular microarchitecture and has been shown to improve fracture risk stratification independently of BMD [2,3,4,5,6,7,8]. In clinical practice, the combination of preserved BMD and degraded TBS may identify imaging discordance that is not captured by densitometric classification alone. Whether this discordance translates into higher fracture risk requires confirmation in studies including fracture history, FRAX probability, and longitudinal fracture outcomes.

### 4.5. Methodological Considerations Regarding TBS and BMI

The interpretation of TBS in patients with higher BMI requires particular methodological caution. Soft tissue thickness and body habitus may influence lumbar spine DXA image texture and, consequently, TBS values. This issue has been recognized in the development of updated TBS approaches, including TBS version 4.0, which incorporates direct soft-tissue thickness correction; in the present study, TBS values were extracted from clinical reports generated with TBS iNsight software version 3.1.2. Therefore, the direct soft-tissue thickness correction implemented in TBS version 4.0 was not applied.

Consequently, the observed inverse association between BMI and TBS should not be interpreted exclusively as evidence of biological trabecular deterioration. It may reflect obesity-related skeletal effects, technical influences related to soft tissue thickness, or a combination of both. This uncertainty is particularly relevant in obesity research and supports a cautious interpretation of lower TBS values in patients with higher BMI.

Nevertheless, the clinical relevance of the BMI–TBS relationship remains important. From the clinician’s perspective, the DXA/TBS report reflects the combined output of patient characteristics, acquisition conditions, and software-derived interpretation. If higher BMI is consistently associated with lower reported TBS despite higher BMD, this discordance remains relevant for real-world interpretation. The present study therefore supports a cautious but practical approach: in overweight and obese postmenopausal women, BMD and TBS should be interpreted together, and discordant findings should prompt more nuanced skeletal risk assessment.

### 4.6. Strengths

This study has several strengths. First, it used a real-world DXA/TBS cohort of postmenopausal women, reflecting routine clinical practice rather than a highly selected research population. Second, the analysis was based on a predefined data lock and explicit inclusion and exclusion criteria. Third, bilateral hip measurements were retained when available, and minimum hip values were used to reduce the risk of underestimating densitometric impairment. Fourth, the study focused on a distinct and clinically relevant question: the divergent relationship of BMI with BMD and TBS. Finally, the analysis included both continuous associations and clinically interpretable discordant phenotypes, improving the translational relevance of the findings.

### 4.7. Limitations

The study also has limitations. Its retrospective and single-center design limits causal inference and generalizability. The sample size was moderate, and subgroup analyses across BMI categories should be interpreted cautiously. The limited sample size is particularly relevant for subgroup analyses. The obesity subgroup included only 33 participants, and several discordant phenotype analyses involved small cell counts. Consequently, subgroup prevalence estimates may be unstable, as reflected by the relatively wide confidence intervals, and these findings should be interpreted as exploratory and hypothesis-generating rather than definitive. The analysis was based on retrospective DXA/TBS reports, and several clinically important variables were not systematically available, including previous fragility fractures, osteoporosis treatment, glucocorticoid use, diabetes mellitus, vitamin D status, calcium supplementation, smoking, alcohol consumption, physical activity, rheumatoid arthritis or other inflammatory diseases, renal function, fall history, and other relevant comorbidities. These variables may influence both BMD and TBS and may partially explain the observed associations. For example, diabetes mellitus, glucocorticoid exposure, inflammatory disease, vitamin D deficiency, anti-osteoporotic treatment, and differences in physical activity may affect bone density, trabecular texture, or both. Therefore, residual confounding cannot be excluded, and the multivariable models should be interpreted as partially adjusted association models rather than as evidence of independent causal effects of BMI on TBS.

Another important limitation is that BMI was used as the anthropometric exposure variable, although it does not distinguish between fat mass, lean mass, visceral adiposity, fat distribution, or sarcopenic obesity. Although DXA can provide body composition parameters, these outputs were not systematically available in the retrospective clinical DXA/TBS reports included in the present analysis. Therefore, the study could not evaluate whether fat mass, lean mass, android/gynoid fat distribution, or visceral adiposity estimates were more closely associated with BMD or TBS than BMI. This limitation is clinically relevant because individuals with similar BMI may have markedly different body composition profiles, which may influence densitometric and DXA-derived trabecular texture parameters in different ways. Future studies incorporating DXA-derived body composition analysis are needed to clarify the relative contribution of adiposity, lean mass, and fat distribution to BMD–TBS discordance.

Finally, TBS interpretation in patients with obesity requires caution because soft tissue thickness may influence TBS values. Although TBS remains clinically useful as a complementary DXA-derived parameter, the potential technical influence of body habitus should be considered when interpreting lower TBS in patients with high BMI [28,42,43].

## 5. Conclusions

In postmenopausal women, higher BMI was associated with higher BMD but lower TBS, indicating a divergent relationship between densitometric findings and DXA-derived trabecular texture parameters. Given the potential influence of soft tissue thickness on TBS, this finding should be interpreted cautiously, particularly in patients with obesity. Patients with obesity more frequently showed imaging-defined discordant skeletal phenotypes characterized by non-osteoporotic DXA findings combined with degraded TBS. These findings support the complementary interpretation of TBS alongside BMD, particularly in overweight and obese postmenopausal women. However, because fracture outcomes and several clinically relevant confounders were not available, the present results should be interpreted as imaging-based associations rather than direct evidence of clinical underdiagnosis or misclassification. Larger studies with detailed clinical risk profiles and longitudinal fracture data are needed to determine the prognostic significance of these discordant skeletal phenotypes.

## Figures and Tables

**Table 1:** Baseline and densitometric characteristics stratified by BMI category.

Variable	Total (n = 106)	Under/Normal-Weight (n = 36)	Overweight (n = 37)	Obesity (n = 33)	*p* Value
Age, years	65.2 ± 7.6	64.5 ± 8.8	64.5 ± 6.5	66.7 ± 7.4	0.385
Height, cm	159.3 ± 6.8	158.5 ± 7.6	159.9 ± 6.5	159.4 ± 6.2	0.678
Weight, kg	69.4 ± 13.2	56.2 ± 6.3	70.1 ± 7.6	83.1 ± 8.8	<0.001
BMI, kg/m^2^	27.3 ± 4.5	22.3 ± 1.8	27.3 ± 1.5	32.6 ± 1.9	<0.001
Age at menopause, years	47.6 ± 5.6	47.1 ± 6.2	47.4 ± 4.7	48.5 ± 5.7	0.516
Years since menopause	17.5 ± 8.3	17.4 ± 8.7	17.1 ± 8.0	18.2 ± 8.3	0.867
Lumbar spine BMD, g/cm^2^	0.807 ± 0.134	0.768 ± 0.128	0.788 ± 0.091	0.870 ± 0.160	0.003
Lumbar spine T-score	−1.8 ± 1.9	−2.0 ± 2.2	−2.0 ± 1.5	−1.2 ± 1.8	0.133
Minimum femoral neck BMD, g/cm^2^	0.641 ± 0.100	0.611 ± 0.091	0.630 ± 0.075	0.687 ± 0.119	0.004
Minimum femoral neck T-score	−1.9 ± 0.9	−2.1 ± 0.8	−2.0 ± 0.7	−1.5 ± 1.1	0.004
Minimum total hip BMD, g/cm^2^	0.793 ± 0.122	0.738 ± 0.120	0.777 ± 0.093	0.870 ± 0.116	<0.001
Minimum total hip T-score	−1.1 ± 1.1	−1.5 ± 1.3	−1.3 ± 0.7	−0.6 ± 0.9	<0.001
Trabecular Bone Score	1.247 ± 0.099	1.283 ± 0.084	1.255 ± 0.088	1.199 ± 0.109	0.001
Overall DXA category: Normal	5 (4.7)	0 (0.0)	1 (2.7)	4 (12.1)	0.012
Osteopenia	47 (44.3)	11 (30.6)	18 (48.6)	18 (54.5)	
Osteoporosis	54 (50.9)	25 (69.4)	18 (48.6)	11 (33.3)	
TBS category: Normal	29 (27.4)	12 (33.3)	12 (32.4)	5 (15.2)	0.118
Partially degraded	33 (31.1)	14 (38.9)	10 (27.0)	9 (27.3)	
Degraded	44 (41.5)	10 (27.8)	15 (40.5)	19 (57.6)	
Bone Resilience Index category: Normal	2 (1.9)	0 (0.0)	1 (2.7)	1 (3.0)	0.759
Normal/Sufficient	1 (0.9)	0 (0.0)	0 (0.0)	1 (3.0)	
Moderate	30 (28.3)	10 (27.8)	11 (29.7)	9 (27.3)	
Low	66 (62.3)	25 (69.4)	22 (59.5)	19 (57.6)	
Severely low	7 (6.6)	1 (2.8)	3 (8.1)	3 (9.1)	

**Table 2:** Pearson correlations between BMI and bone parameters.

Parameter Associated with BMI	n	Pearson r	95% CI	*p* Value
Trabecular Bone Score	106	−0.307	−0.470 to −0.123	0.001
Lumbar spine BMD	106	0.392	0.218 to 0.542	<0.001
Lumbar spine T-score	106	0.196	0.005 to 0.373	0.044
Minimum femoral neck BMD	106	0.382	0.206 to 0.534	<0.001
Minimum femoral neck T-score	106	0.379	0.203 to 0.532	<0.001
Minimum total hip BMD	106	0.508	0.352 to 0.637	<0.001
Minimum total hip T-score	106	0.391	0.216 to 0.541	<0.001
Lowest hip T-score	106	0.394	0.219 to 0.544	<0.001
Age	106	0.051	−0.141 to 0.239	0.605
Years since menopause	106	0.015	−0.176 to 0.206	0.875

Notes: Pearson r values are shown with Fisher-z 95% confidence intervals. BMD = bone mineral density; TBS = Trabecular Bone Score.

**Table 3:** Linear regression models for Trabecular Bone Score.

Predictor	Model 1 β (95% CI); *p*	Model 2 β (95% CI); *p*	Model 3 β (95% CI); *p*
BMI	−0.0068 (−0.0108 to −0.0027); 0.001	−0.0066 (−0.0107 to −0.0026); 0.002	−0.0126 (−0.0163 to −0.0089); <0.001
Age	—	−0.0010 (−0.0047 to 0.0027); 0.592	−0.0010 (−0.0042 to 0.0021); 0.527
Years since menopause	—	−0.0014 (−0.0047 to 0.0020); 0.430	0.0001 (−0.0027 to 0.0029); 0.958
Lumbar spine BMD	—	—	0.389 (0.247 to 0.530); <0.001
Lowest hip T-score	—	—	0.017 (−0.006 to 0.040); 0.139
Model R^2^	0.094	0.126	0.436
Adjusted R^2^	0.085	0.100	0.408
Model *p* value	0.001	0.003	<0.001

Notes: Outcome variable: Trabecular Bone Score. β values are unstandardized regression coefficients with 95% confidence intervals. Model 1: BMI only. Model 2: BMI, age, and years since menopause. Model 3: BMI, age, years since menopause, lumbar spine BMD, and lowest hip T-score.

**Table 4:** TBS impairment and discordant skeletal phenotypes across BMI categories.

Phenotype or TBS Category	Total (n = 106)	Under/Normal-weight (n = 36)	Overweight (n = 37)	Obesity (n = 33)	χ^2^ *p* Value	Trend *p* Value
Degraded TBS	44 (41.5)	10 (27.8)	15 (40.5)	19 (57.6)	0.042	0.014
Partially degraded or degraded TBS	77 (72.6)	24 (66.7)	25 (67.6)	28 (84.8)	0.165	0.098
Low or severely low Bone Resilience Index	73 (68.9)	26 (72.2)	25 (67.6)	22 (66.7)	0.864	0.615
Overall DXA non-osteoporotic +degraded TBS	19 (17.9)	2 (5.6)	6 (16.2)	11 (33.3)	0.010	0.005
Hip DXA non-osteoporotic +degraded TBS	32 (30.2)	5 (13.9)	10 (27.0)	17 (51.5)	0.003	0.001
Overall DXA non-osteoporotic + partially degraded/degraded TBS	31 (29.2)	4 (11.1)	9 (24.3)	18 (54.5)	<0.001	<0.001
Hip DXA non-osteoporotic + partially degraded/degraded TBS	53 (50.0)	14 (38.9)	15 (40.5)	24 (72.7)	0.007	0.007

Notes: Values are n (%). χ^2^ *p* values are from Pearson chi-square tests across BMI categories. Trend *p* values are from logistic regression using ordinal BMI category. For the full ordinal TBS category distribution, Spearman rho = 0.238, *p* = 0.014.

## Data Availability

The data supporting the findings of this study are available from the corresponding author upon reasonable request, subject to institutional and ethical restrictions.
